# Microscopic hematuria in IgA nephropathy: a biomarker of disease activity

**DOI:** 10.1093/ckj/sfag003

**Published:** 2026-01-12

**Authors:** Jürgen Floege, Fernando C Fervenza, Rosanna Coppo

**Affiliations:** Division of Nephrology and Rheumatology, RWTH Aachen University Hospital, Aachen, Germany; Division of Nephrology and Hypertension, Mayo Clinic, Rochester, MN, USA; Fondazione Ricerca Molinette, Turin, Italy

**Keywords:** CKD, ESRD, hematuria, IgA nephropathy, inflammation

## Abstract

Immunoglobulin A nephropathy (IgAN) is an immune-mediated disease of B-cell origin and the most common primary glomerulonephritis worldwide, which often progresses to kidney failure within 10–20 years of diagnosis. Microscopic hematuria is frequently observed in IgAN; it is thought to result from damage to the glomerular filtration barrier caused by pathogenic immune complex deposits, allowing red blood cells to leak into the urinary space. Emerging evidence suggests that microscopic hematuria in IgAN may be linked to active glomerular inflammation, poorer disease prognosis and progressive kidney function decline. Despite this, it remains an underutilized biomarker for IgAN because of a lack of standardization (which can lead to preanalytical errors), challenging logistical considerations in large multicenter trials and non-glomerular hematuria as a confounding factor. The “proteinuria-centric” approach by the nephrology community may overlook that active forms of glomerulonephritis manifest with both proteinuria and hematuria, in contrast to primary podocytopathies where proteinuria is the main feature. When properly assessed, microscopic hematuria is a prognostically relevant biomarker in IgAN that may improve risk stratification and assessment of therapeutic response when evaluated alongside traditional biomarkers. This review evaluates the methods of assessment, pathophysiology and clinical utility of microscopic hematuria in IgAN.

## INTRODUCTION

Microscopic hematuria [presence of red blood cells (RBC) in the urine without visible blood] is a common but nonspecific clinical presentation of glomerulonephritis in general, but particularly in immunoglobulin A nephropathy (IgAN) [1, 2]. IgAN is the most common primary glomerulonephritis globally, and a leading cause of chronic kidney disease (CKD) in young adults [3, 4]. IgAN poses a significant challenge because of its progressive nature and impact on both life expectancy and quality of life [5–7]. While estimates of IgAN incidence range from 0.06 to 10.5 per 100 000 [8], the true incidence remains uncertain as definitive diagnosis relies on kidney biopsy—a requirement that likely contributes to underdiagnosis.

Initial disease presentation is often asymptomatic or nonspecific, and the presence of microscopic hematuria is often overlooked. As a result, the majority of patients already have signs of CKD at definitive diagnosis [2, 6, 9]. Over the past 30 years, the age at diagnosis has increased, likely because of increasing thresholds for kidney biopsy due in part to historically limited treatment options specific for IgAN [beyond renin–angiotensin–aldosterone system inhibitors (RAASi), blood pressure control and lifestyle management], leading to a lower impetus for an invasive biopsy procedure. Such delays fail to address that early disease-modifying intervention may reduce lifetime risk for kidney failure in patients at high risk for progression [7]. Delays in diagnosis likely contribute to the observation that by the time of IgAN diagnosis up to 40% of patients with IgAN have stage 3 CKD, 17.9% have stage 4 CKD, and 11.2% have stage 5 CKD (kidney failure) [6]. Furthermore, observations from the UK National Registry of Rare Kidney Diseases (RaDaR) IgAN cohort [*n* = 2439; biopsy-proven IgAN with proteinuria >0.5 g/day or estimated glomerular filtration rate (eGFR) <60 mL/min] demonstrated that nearly all patients were at risk of kidney failure within their lifetime, regardless of age or eGFR at diagnosis [7]. These diagnostic gaps underscore the need for scalable, reproducible biomarkers to enable earlier detection and risk stratification in routine care.

Traditional prognostic markers in IgAN include proteinuria, eGFR decline and histological findings [10]. Although not specific to IgAN, hematuria is the most common clinical manifestation, reported in the majority (70%–100%) of patients with IgAN [11, 12]. Hematuria may result from structural alteration of the glomerular basement membrane (e.g. Alport disease); however, it may also reflect active inflammation triggered by the pathogenic mechanisms that drive IgAN disease progression [13, 14]. As isolated microscopic hematuria may occur in IgAN (i.e. asymptomatically before the development of overt proteinuria or impaired eGFR) [15, 16], hematuria assessment may offer the potential for earlier detection of active disease that requires intervention. Despite the mechanistic link and clinical presentation, hematuria is not widely used for IgAN risk stratification and remains a largely underutilized disease biomarker [11, 17]. Historically, sample preparation and handling errors, lack of standardization and the inherent day-to-day variability of microscopic hematuria have limited its inclusion in clinical practice and its use as a prognostic marker in large multicenter studies [17]. However, assessment by alternative and sensitive methods, such as detection of hemoglobinuria, may offer benefits in further confirming the presence of hematuria or in raising the possibility of a false-negative urinalysis when confronted with a urinalysis reporting <3 RBC per high-power field (HPF) but ≥1+ hemoglobinuria via dipstick.

This review focuses on the pathophysiology and clinical significance of microscopic hematuria as a marker of glomerular inflammation and prognostic indicator in IgAN, as it is observed in early phases of IgAN as well as in later stages when the disease is fully expressed with proteinuria and a decrease in eGFR [11]. Methods to assess microscopic hematuria, and the limitations of their implementation, will be considered. Changes in microscopic hematuria following treatment will be described, to highlight its clinical utility as a marker of disease activity and response to immunosuppressive therapy in IgAN.

## DIAGNOSIS AND ASSESSMENT OF MICROSCOPIC GLOMERULAR HEMATURIA AND LIMITATIONS TO ITS IMPLEMENTATION

While glomerular hematuria is indicative of a disruption in normal filtration and barrier function at the glomerulus, it is important to keep in mind that bleeding may occur at any point from the glomerulus to the urethra [18], resulting from malignancy (most commonly bladder cancers), medication (cyclophosphamide), gynecological conditions, infection, inflammation, urinary tract calculi, benign prostatic hyperplasia, and congenital or anatomic abnormalities [19]. Thus, it is important for the treating nephrologist to consider these diagnoses at least in a subgroup of patients with IgAN who meet certain age-related or other criteria even before kidney biopsy and the actual diagnosis of IgAN. Furthermore, transient causes of hematuria, such as menstruation, or exercise-induced hematuria or myoglobinuria and rhabdomyolysis, must be ruled out [20]. Glomerular hematuria becomes more likely if accompanied by hypertension, history of kidney disease, dysmorphic RBC, red blood cell casts, higher grades of proteinuria or reductions in eGFR [19].

Several methodologies are available for the assessment and diagnosis of hematuria. Initial detection is often via dipstick urinalysis [21] owing to its ease of use and accessibility. This chemical analysis kit allows indirect grading of samples [22] by the detection of hemoglobin peroxidase activity [23]. Dipstick urinalysis cannot distinguish between hemoglobin from intact or lysed RBC (due to degradation of the urine sample through improper storage or inefficient processing timelines) and detects the peroxidase activity of myoglobin as a confounding false positive [19, 23, 24]. While considering these potential limitations of the assay, determination of hemoglobinuria by dipstick provides an excellent alternative indirect marker for hematuria, in particular given that “true” glomerular hemoglobinuria, for example in hemolysis, is very rare. Use of automated dipstick test strip analyzers can enable grading into negative/trace [hemoglobin (Hb): ≤0.03 mg/dL], +1 (Hb: 0.06 mg/dL), +2 (Hb: 0.15 mg/dL) and +3 (Hb: 0.75 mg/dL) hematuria [25]. The accuracy of dipstick urinalysis has shown strong correlation with microscopy when detecting microscopic hematuria ≥3 RBC/HPF [22].

Confirmatory testing of dipstick-positive urine by HPF microscopy is advised in current guidelines [21], but urinalysis must be performed by analyzing a fresh urine sample, with a requirement for ready access to a urinary microscope or automated urine analyzer. Clinical thresholds for clinically significant IgAN-related hematuria based on measurement of RBC by microscopy remain under discussion [17, 26]. Although there are no formally established definitions for microscopic hematuria severity, published criteria include absent (<3 RBC/HPF), mild (3–10 RBC/HPF), moderate (11–20 RBC/HPF) and severe/significant (≥21 to ≥30 RBC/HPF) hematuria [13, 17, 27, 28]. Other studies have stratified hematuria increases by smaller increments [13] or advised alignment of definitions based on the detection method [17].

Beyond these traditional diagnostic methods, the automation of urine microscopy and flow cytometry has promoted faster and more efficient processing of samples, with the caveat that the samples might need to be shipped to an analytical laboratory, thereby delaying the analysis. Comparison of manual and automated urinalysis in the detection of microscopic hematuria in patients with IgAN has shown strong correlation between methods (r = 0.948; *P* < .001) [29]; however, institutional validation and calibration of automated methodologies are needed [30].

Urinalysis is prone to preanalytical and sampling errors, affecting interpretation and diagnosis [24]. Awareness of these risks and implementation of preventative measures is key to improving reliability of urinalysis. As with most biological matrices, urine is labile and its composition changes soon after voiding; accurate handling and storage are crucial to the integrity of its analysis [31]. Ideally, urinalysis should be conducted within 90 min post-collection; otherwise, samples should be stored at 4°C to slow the rate of degradation and tested within 24 h [31, 32]. This time sensitivity could result in missed diagnoses of hematuria, especially if the initial count was close to the diagnostic threshold [33].

**Figure 1:**
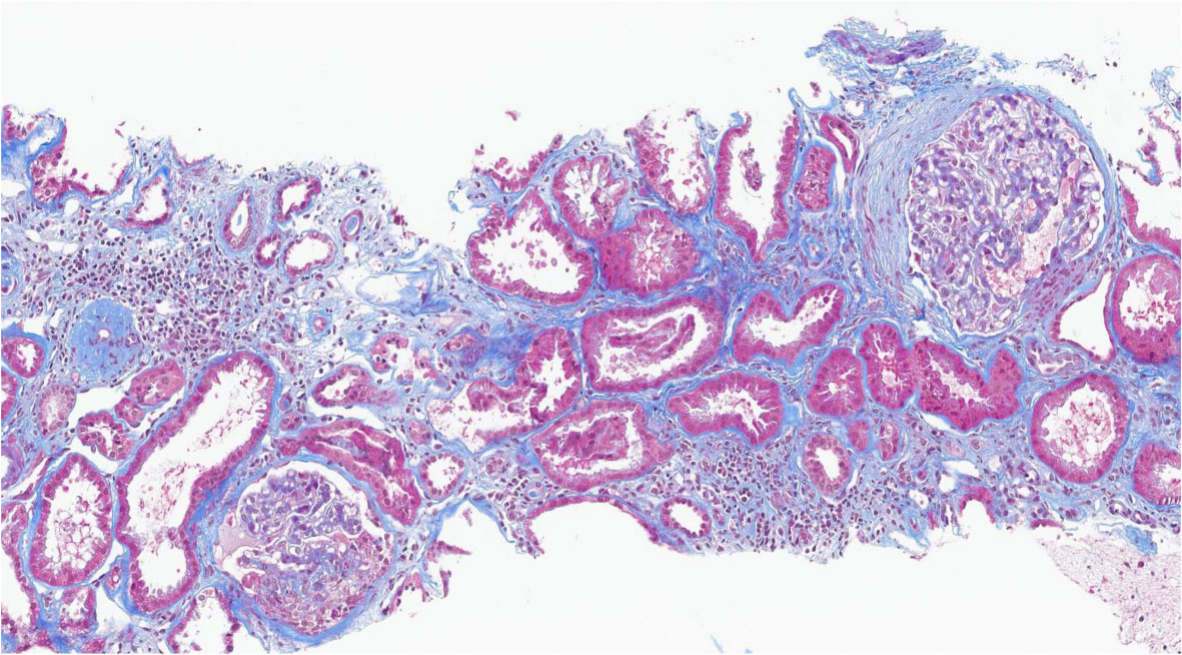
Kidney biopsy of IgAN patient presenting with negative/trace hematuria with positive (1+) hemoglobinuria. A 30-year-old female patient, with IgAN diagnosed in 2017 (M1, E0, S1, T0, C0), serum creatinine 0.8 mg/dL, urine protein:creatinine ratio 3.7 g/g and positive hematuria as assessed by microscopy (5–10 RBC/HPF). The patient was treated per Pozzi protocol (6-month corticosteroid using pulse intravenous methylprednisolone and oral prednisone); urinalysis remained active and dapagliflozin was added in 2021. In 2024, the patient presented with serum creatinine 1.3 mg/dL, quantified 24 h creatinine clearance of 54 mL/min/1.73 m^2^ and proteinuria 2 g/24 h. Urinalysis on a fresh urine sample showed <3 RBC/HPF, but with a positive urine dipstick reading of 1+. The presence of hemoglobinuria superseded the negative hematuria to warrant a repeat biopsy. A second kidney biopsy, depicted here, shows crescentic IgAN.

Importantly, assessment by microscopy or cytometry may detract from the fact that it is the presence of any hematuria/hemoglobinuria signal that is key, regardless of its degree. In the proper context, the presence of hemoglobinuria in a patient with negative urinalysis should prompt a new urinalysis using a fresh urine sample and should serve as an indication of active glomerular inflammation in a patient with declining kidney function, as described in Fig. 1.

A lack of agreement on standardized methodologies and protocols poses challenges for data interpretation across studies. While the use of a centralized analytical laboratory enables standardization, it may inadvertently hinder participation in multicenter and international clinical trials, due to logistical constraints. Overlapping variations in definition of remission and degrees of severity of microscopic hematuria, coupled with inconsistent reporting of methods, can create uncertainty when comparing therapeutic efficacies in IgAN. That is why comparing patients as having hematuria/hemoglobinuria vs no hematuria/hemoglobinuria provides a practical approach, devoid of the issues of hematuria quantification, to access therapeutic interventions and has been implemented in recent clinical trials [34, 35].

Finally, it should be noted that microscopic hematuria can be stable or can vary from day to day. As such, several studies have emphasized the value of monitoring hematuria levels over time, with remission/disappearance of microscopic hematuria defined as <5 RBC/HPF [36] and persistence as longer than 4–6 weeks of >5 RBC/HPF [37].

## PATHOPHYSIOLOGY OF MICROSCOPIC HEMATURIA IN IGAN

In CKD, glomerular hematuria is a result of structural and functional disruptions within the glomerular filtration barrier of the kidney [38]. This trilayered barrier of fenestrated endothelial cells, glomerular basement membrane and podocyte foot processes provides both size and charge selectivity. This selectivity prevents the passage of negatively charged plasma protein and of RBC and their breakdown products into the urinary space [39]. Pathological hematuria occurs when insults (genetic, metabolic and/or immunologic) compromise one or more of these layers [38, 40]. In immune-mediated damage, mesangial deposition of immune complexes stimulates proliferation of mesangial cells and inflammation, including an increase in glomerular collagenase activity, which leads to alteration in glomerular barrier integrity [40, 41]. Although hyperfiltration and structural changes to the glomerular filtration barrier result in increased passage of proteins into the urinary space, active uptake of proteins via receptor-mediated endocytosis in the proximal tubule may permit the reabsorption of glomerular filtrated proteins, with the capacity to reabsorb 1.9 g albumin and 9.6 g of low molecular weight proteins per day [42]. Unlike active tubular reabsorption of protein, there are no reported mechanisms that permit reuptake of RBC.

**Figure 2:**
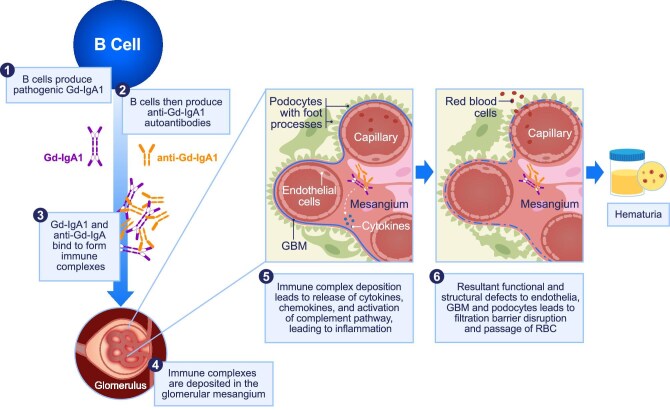
Pathophysiology of hematuria in IgAN. In individuals with IgAN, B cells respond to key cytokines, BAFF and APRIL, to produce pathogenic Gd-IgA1, which triggers production of anti-Gd-IgA1 autoantibodies. Gd-IgA1 and anti-Gd-IgA1 bind to each other, forming immune complexes. Deposition of Gd-IgA1/anti-Gd-IgA1 immune complexes in the kidney elicits release of cytokines and chemokines and activation of the complement cascade, which causes inflammation and leads to injury of glomerular capillary endothelial cells, podocytes and the glomerular basement membrane, permitting passage of RBC into the urinary space. GBM, glomerular basement membrane.

The role of IgAN pathophysiology (four-hit hypothesis) and glomerular inflammation in the development of hematuria has been described previously in detail (Fig. 2) [14, 17, 43]. Briefly, B cells respond to key cytokines B-cell Activating Factor (BAFF) and A PRoliferation Inducing Ligand (APRIL) to produce pathogenic forms of IgA [including galactose-deficient IgA1 (Gd-IgA1); HIT 1] and its autoantibodies (anti-Gd-IgA1; HIT 2). These pathogenic antibodies and autoantibodies then bind to each other to form pathogenic immune complexes (HIT 3). These immune complexes accumulate in the mesangium (HIT 4) [44]. Experimental models show that when IgA is overproduced or when exogenous Gd-IgA1 immune complexes are administered to mice, increased glomerular deposition of pathogenic immune complexes leads to activation of complement pathways (alternative and lectin) that drive glomerular inflammation, and is associated with hematuria [45–47]. Furthermore, in a murine model of IgAN, hematuria, but not proteinuria, was decreased following reductions in serum and mesangial IgA after 1 week of administration of an IgA protease [48].

Activation of inflammatory pathways contributes to podocyte and endothelial injuries, which facilitate RBC passage into the urinary space (Fig. 2) [17, 49]. Moreover, peripheral blood mononuclear cells from patients with IgAN respond to lipopolysaccharide to a greater extent than those from healthy individuals by enhancing chemokine receptor expression, indicating an upregulation of cytotoxic effector lymphocytes in IgAN [50]. These results further suggest that the contribution of infiltrating pro-inflammatory lymphocytes may modify the glomerular filtration barrier [14]. Further cellular insult may occur in individuals with hematuria, where lysis of RBC in the urinary space releases hemoglobin and heme-related products, which are taken up by tubular cells. This uptake results in oxidative damage, tubulointerstitial fibrosis, podocyte dysfunction, apoptosis and podocyte detachment from glomerular capillaries [14, 17]. Heme-related products may further elicit pro-inflammatory events such as reactive oxygen species generation, increased lipid peroxidation, cytokine release, leukocyte activation and migration, and various inflammatory signaling cascades [14, 51]. Persistent hematuria and tubular exposure to heme proteins contribute to chronic inflammation in the kidney [52]. Indeed, repeated administration of hemoglobin produces chronic tubulointerstitial inflammation in animal models [53]. Based on this evidence, hematuria may be both a marker and a mediator of the pathology of glomerulonephritis. Recent data on urinary proteomic profiling using the IgAN237 classifier indicate that urinary proteomics captures structural remodeling associated with progressive loss of kidney function in patients with IgAN [54]. The value of the urinary peptide profile in addition to microscopic hematuria represents a promising area for future research.

## CLINICAL SIGNIFICANCE OF HEMATURIA IN IGAN

Historically, reducing proteinuria has been the primary treatment goal in IgAN. As such, hematuria has been relatively overlooked in terms of its relationship with disease progression and its potential value as a biomarker for disease activity [11, 17]. While some studies have shown no association of hematuria with kidney failure or long-term kidney survival [55–57], others have shown that persistent or time-averaged increases in microscopic hematuria are associated with eGFR decline, CKD progression and poorer prognosis in patients with IgAN (Table 1) [1, 13, 26, 28, 29, 58]. In a meta-analysis of 13 studies with a total of 5660 patients with IgAN, initial microscopic hematuria observed at time of biopsy or apparent disease onset was associated with an 87% increase in kidney failure risk [risk ratio (RR) 1.87; 95% confidence interval (CI) 1.40–2.50; *P* < .001]. Additionally, persistence of hematuria has been identified as a potential independent risk factor for poor kidney outcomes (kidney failure or a 50% decline in eGFR) [59], and may enhance risk prediction alongside proteinuria and eGFR.

**Table 1:** Summary of key studies on IgAN and hematuria.

Study	Population	Definition of hematuria	Renal outcome	Risk of progression	Association with proteinuria	Impact on eGFR decline	Conclusions
Goto *et al*., 2009 [28]	*N* = 790; biopsy-proven IgAN	Mild hematuria = 1–29 RBC/HPF	Doubling of serum creatinine within 10 years	Multivariate logistic regression analysis; OR 2.3 (95% CI 1.2–4.3)	N/A	N/A	Mild hematuria was an independent risk factor for loss of renal function
Iwasaki *et al*., 2016 [55]	*N* = 75; biopsy-proven IgAN^a^; proteinuria >1 g/day	Low = ≤20 RBC/HPF; high = >20 RBC/HPF	ESKD	Univariate Cox regression analysis; HR 0.75 (95% CI 0.55–1.00; *P* = .0529)	No difference between low and high groups at baseline, 1 year or 2 years	No difference between low and high groups at baseline, 1 year or 2 years	Higher degree of hematuria was not associated with risk of ESKD
Tanaka *et al*., 2015 [56]	*N* = 88; biopsy-proven IgAN^b^; proteinuria <0.5 g/day	Low = <20 RBC/HPF; high = ≥20 RBC/HPF	15-year renal survival rate	15-year renal survival per Kaplan–Meier method. Low group: 83.4%; high group: 100%. Log-rank test: *P* = .201	No difference between groups at time of biopsy or at 1 year, 2 years, 3 years, 4 years or 5 years	N/A	Hematuria was not associated with long-term kidney survival
Ebbestad *et al*., 2022 [57]	*N* = 77; biopsy-proven IgAN with high-degree hematuria (>10 RBC/HPF, or urine dipstick grading of 2–3)	Microscopic hematuria per urine dipstick:^c^ low = 0–1; high = 2–3	Composite outcome of 50% decrease in eGFR from baseline or ESKD	Univariate Cox regression analysis. High-grade microhematuria not associated with renal outcome (*P* = .144)	TA-albuminuria 5 years post-biopsy (mean ± SD; g/day). Low = 0.55 ± 0.69; high = 0.97 ± 1.54 (*P* = .145)	eGFR slope 5 years post-biopsy (mean ± SD; mL/min/year). Low = 0.05 ± 3.75; high = -2.20 ± 5.18 (*P* = 0.103)	High degree of microscopic hematuria did not have a significant impact on renal outcomes
Yu *et al*., 2020 [29]	*N* = 1333; biopsy-proven IgAN	Remission: 6-month TA manual count of ≤5 RBC/HPF, and automated count of ≤28 RBC/μL. Persistent: TA manual count of >5 RBC/HPF, and automated count of >28 RBC/μL	Composite progression event 50% eGFR decline or ESKD	Multivariate cause-specific hazard model. Persistent hematuria: HR 1.46 (95% CI 1.13–1.87; *P* = .003). Hematuria remission: HR 0.41 (95% CI 0.28–0.61; *P* < .001)	During the first 6 months, hematuria remission improved kidney survival among patients with persistent proteinuria, (HR 0.46; 95% CI 0.32–0.68; *P* < .001), but not among those with proteinuria remission (HR 0.64; 95% CI 0.31–1.29; *P* = .2)	eGFR (mL/min/1.73 m^2^ ± SD) hematuria remission vs persistent: 84.31 ± 30.44 vs 81.65 ± 30.60 (*P* = .2); 50% decline in eGFR (% patients) hematuria remission vs persistent: 11.9% vs 14.8% (*P* = .2)	Persistent hematuria was associated with disease progression and hematuria remission associated with improved renal outcomes
Bobart *et al*., 2021 [13]	*N* = 125; biopsy-proven IgAN with MEST-C scoring who were not on immunosuppressive therapy at biopsy	Severe: ≥21 RBC/HPF. Moderate: 3–20 RBC/HPF. Absent: <3 RBC/HPF	MEST-C scores; time to ESKD; decline in eGFR	Neither baseline nor isolated follow-up microscopic hematuria were associated with ESKD	N/A	Over the follow-up period (mean 3.69 years), degree of hematuria^d^ was associated with an eGFR decline of –1.12 mL/min/1.73 m^2^(95% CI –1.68 to –0.56; *P* = .0001). Severe microscopic hematuria was associated with an increased decline in eGFR (3.99 mL/min/1.73 m^2^; 95% CI –6.94 to –1.06; *P* = .008)	Degree of microscopic hematuria during follow-up is a predictor of eGFR decline after adjusting for clinical and histological parameters
Sevillano *et al*., 2017 [1]	*N* = 112; biopsy-proven IgAN with hematuria at baseline	Persistent TA-hematuria: >5 RBC/HPF. Negative or minimal TA-hematuria: ≤5 RBC/HPF	Renal survival defined by a status free of ESKD; renal function decline >50% over time; eGFR slope	Probability of a clinical event (ESKD or >50% loss of renal function) by Kaplan–Meier. Renal survival was better in patients with negative or minimal hematuria (96.4%, 86.3% and 83.9% for 50%) than in those with persistent hematuria (83.5%, 74.5% and 65.3% for 50%, after 5, 10 and 15 years, respectively, *P* = .01)	Positive trend (NS) between persistent TA-hematuria and TA-proteinuria. Patients with TA-proteinuria >0.75 g/day and persistent hematuria had significantly worse renal survival than those with TA-proteinuria >0.75 g/day and negative/minimal hematuria. TA-proteinuria and TA-hematuria had increased mesangial hypercellularity and worsened renal outcomes (ESKD and >50% reduction in eGFR and rate of renal functional decline) compared with patients without both proteinuria and hematuria	Following hematuria disappearance, rates of renal function decline changed from –6.45 ± 14.66 to –0.18 ± 2.56 mL/min/1.73 m^2^ per year (*P* = .001)	Remission of hematuria may significantly improve IgAN disease outcomes, and hematuria persistence is an independent risk factor for kidney function loss
Huang *et al*., 2023 [26]	*N* = 684; biopsy-proven patients with persistent hematuria	Lower degree <330 RBC/μL. Higher degree ≥330 RBC/μL	MEST-C scores; 30% eGFR decrease or progression to ESKD	Multivariate regression model. Higher-degree hematuria association with 30% eGFR decline (HR 3.93; 95% CI 1.33–11.6; *P* = .01)	No significant difference in proteinuria in those with high- or low-degree hematuria	eGFR of the two cohorts decreased significantly within 10 years of follow-up (*P* < .001), and the eGFR of the higher-degree hematuria cohort decreased even more (*P* = .004)	Patients with high-degree hematuria had poorer pathological manifestations of IgAN with poorer prognosis vs patients with low-degree hematuria
Weng *et al*., 2022 [58]	*N* = 152; biopsy-proven IgAN; microscopic hematuria	Hematuria remission: ≤28 RBC/μL. Persistent microscopic hematuria: >28 RBC/μL	Serum baseline creatinine doubling; presence of ESKD	Persistent microscopic hematuria was associated with lower renal survival rates (*P* = .049). Renal survival rate was the lowest in patients with both persistent hematuria and low TA-serum albumin (*P* = .011)	Patients with remitted hematuria and persistent with proteinuria had a better renal outcome than those with persistence of both hematuria and proteinuria, but a worse outcome than those with remittance of both hematuria and proteinuria (*P* = .001)	Participants with persistent hematuria were more likely to develop ESKD (HR 1.002; 95% CI 1.000–1.001; *P* < .003)	Degree of TA-hematuria is an independent predictor for the progression of IgAN

a Patients were treated with prednisolone 0.8 mg/kg/day for 1 month and tapered to 2.5–5 mg/month; RAASi were added individually.

b Patients had not been treated with corticosteroids or immunosuppressive agents and had not undergone tonsillectomy and were observed for ≥1 year after kidney biopsy.

c For patients whose hematuria was assessed via microscopy, results were converted to urine dipstick outputs based on correlations observed in patients with both microscopy and urine dipstick results: 0–3 RBC/HPF = 0 per urine dipstick, 4–10 RBC/HPF = 1, 10–21 RBC/HPF = 2 and >21 RBC/HPF = 3.

d Degree of hematuria was reported as 0, <3, 3–10, 11–20, 21–30, 31–40, 41–50, 51–100 or >100 RBC/HPF. ESKD, end-stage kidney disease; HR, hazard ratio; N/A, not available; NS, nonsignificant; OR, odds ratio; SD, standard deviation; TAm time averaged.

A seemingly paradoxical observation is that macroscopic hematuria is associated with a 32% decrease in kidney failure risk (RR 0.68; 95% CI 0.58–0.79; *P* < .001) [59]; this may be due to an increased likelihood of seeking medical attention, and rapid clinical referral and consequent follow-up. In addition, it has been proposed that episodes of macroscopic hematuria are most common in children [60] and often rapidly vanish without leaving persistent microscopic hematuria, given the high regenerative capacity in children. Moreover, kidney failure may take much longer to evolve in children (so-called lead-time bias); therefore, such episodes may not have been captured in studies with relatively short (<10-year) follow-up periods.

These findings underscore the importance of screening for hematuria alongside proteinuria for early detection of IgAN prior to the emergence of overt symptoms, as a predictor of progression to kidney failure and for assessing treatment response [1, 17, 29, 36, 43].

Recent evidence suggests that hematuria may be a marker of active glomerular inflammation in general [13, 14, 61]. Studies in patients with kidney involvement in anti-neutrophil cytoplasm antibody-associated vasculitis (granulomatosis with polyangiitis, microscopic polyangiitis or renal-limited vasculitis) reported that the persistence of hematuria may be a sign of low-level disease activity or of risk for potential kidney relapse [62–64]. In a retrospective study of patients with IgAN, microscopic hematuria was significantly associated with three components of the MEST-C [mesangial (M) and endocapillary (E) hypercellularity, segmental sclerosis (S), interstitial fibrosis/tubular atrophy (T), crescents (C)] score (M1, E1 and C ≥1); the latter two may reflect active inflammation in the kidney [13]. These findings gave rise to the hypothesis that hematuria may not only reflect active inflammation but may also indicate response to immune-modulation interventions, supporting hematuria’s potential role as a treatment-sensitive biomarker.

Immunosuppressive treatment has been shown to reduce time-averaged hematuria in patients with IgAN, indicating the role that active inflammation may play in persistent hematuria [1]. Recent recommendations in other forms of glomerulonephritis include the resolution of microscopic hematuria as an inflammation-related treatment goal [65]. This underscores hematuria’s potential prognostic value in IgAN, particularly when used in conjunction with other biomarkers of disease [66–69]. Hematuria, while an independent risk factor for kidney failure [1, 21], may be employed with established markers such as proteinuria to identify high-risk patients who may require more intensive medical monitoring or tailored treatment [38, 70]. However, prospective clinical studies will be required to validate the concept of including hematuria in addition to proteinuria and eGFR when making therapeutic decisions in patients with IgAN. It should be noted that the significance of microscopic hematuria/hemoglobinuria is not related to patients’ eGFR status. We have recently reviewed 514 patients with biopsy-proven IgAN according to urinalysis and kidney biopsy (MEST-C) score and found both eGFR and serum creatinine as poor discriminators for glomerular inflammation (unpublished observations). As such, hematuria/hemoglobinuria alone cannot be used to differentiate early vs late phase of the disease. That is why nephrologists treating these patients need to evaluate hematuria/hemoglobinuria in the context of proteinuria, kidney function and clinical course, and consider a repeat kidney biopsy to ascertain activity in selected patients as illustrated by the case presented in Fig. 1.

## HEMATURIA AS AN INDICATOR OF THERAPEUTIC RESPONSE IN IGAN

The current first-line standard of care for patients with IgAN includes nonspecific CKD therapy consisting of RAASi or dual endothelin angiotensin receptor antagonists, and sodium-glucose co-transporter-2 inhibitors (SGLT2i) [10, 21]. RAASi have been shown to reduce proteinuria, but no studies so far have reported hematuria reduction associated with response to RAASi therapy. Reductions in hematuria have been observed with recently approved therapies (complement inhibitors and targeted-release budesonide) and emerging therapies (B-cell modulators) (therapies summarized in Table 2). Trials in patients with IgAN have demonstrated modest reductions in hematuria with factor B inhibition (hematuria remission in 38.7% of participants receiving iptacopan vs 16.3% receiving placebo after 9 months’ treatment) [71] and with targeted-release budesonide (with no hematuria reported in 59.5% of participants receiving targeted-release budesonide vs 38.8% receiving placebo for 9 months, with a 15-month follow-up period) [72]. The impact of these treatments is likely due to their broad anti-inflammatory effects and the mechanistic relationship between inflammation and hematuria.

**Table 2:** Summary of the effect of IgAN treatments on hematuria and other renal outcomes.

Study, NCT number, data references	IgAN treatment	Mechanism of action	Population	Effect on renal outcome	Definition of hematuria	Effect on hematuria
Approved therapies						
Prospective uncontrolled trial, Dong *et al*., 2023 [81]	Dapagliflozin or canagliflozin	SGLT2i	*N* = 93; biopsy-proven IgAN	After 3 months of SGLT2i therapy, a significant reduction in proteinuria of 22.9% (95% CI 16.1–29.7%; *P* < .001) was observed compared with baseline	Not defined	The number of RBC/HPF decreased after 6 months of SGLT2i treatment compared with baseline (8.9 vs 12.3; *P* = .01)
APPLUSE-IgAN (NCT04578834), randomized, DBPC, Perkovic *et al*., 2025 [71]	Iptacopan	Complement factor B inhibitor	*N* = 443; biopsy-proven IgAN	At Month 9, the adjusted geometric mean 24-h UPCR was 38.3% (95% CI 26.0–48.6%; two-sided *P* < .001) lower with iptacopan than with placebo	Urine dipstick ≥1+	Among patients who had hematuria at baseline, hematuria was no longer present at Month 9 in 38.7% (95% CI 28.8–49.4%) of those receiving iptacopan and in 16.3% (95% CI 9.2–25.8%) of those in the placebo group
NefIgArd (NCT03643965), randomized, DBPC, Zhang *et al*., 2024 [72]	Nefecon	Targeted-release corticosteroid	*N* = 364; biopsy-proven IgAN	Time-weighted average eGFR improvement over 2 years within the global population of 5.1 mL/min/1.73 m^2^ vs placebo (95% CI 3.2–7.4; *P* < .001). 41% reduction in TA UPCR vs placebo between 12 and 24 months (*P* < .001)	Not defined	Proportion of patients with microscopic hematuria decreased from 66.5% at baseline to 40.5% during follow-up, compared with a decrease from 67.8% to 61.2% in the placebo group
Emerging therapies						
ORIGIN 2b (NCT04716231), randomized, DBPC, Lafayette *et al*., 2024 [73], Barratt *et al*., 2024 [74]^a^, Floege *et al*., 2024 [75]^a^	Atacicept	BAFF and APRIL inhibitor	*N* = 116; biopsy-proven IgAN (UPCR, eGFR); *n* = 67; hematuria	At 36 weeks, atacicept reduced UPCR compared with placebo (35%; *P* = 0.012). eGFR increased from baseline by 1% with atacicept compared with an 8% reduction in the placebo group: an 11% difference (*P* = .022)	Urine dipstick ≥1+	At 36 weeks, hematuria improved to negative/trace in most participants receiving atacicept 150 mg [80% (12/15) vs 5% placebo (1/19)] with hematuria 1+ or higher at baseline. Gd-IgA1 reduction was correlated with lower hematuria at Week 36 (r = 0.35; *P* = .0003)
ORIGIN 2b (NCT04716231), open-label extension, Barratt *et al*., 2025 [76]	Atacicept	BAFF and APRIL inhibitor	*N* = 113; biopsy-proven IgAN	Atacicept reduced proteinuria with a reduction of –52% from baseline at 96 weeks, with a mean eGFR annualized slope of –0.6 mL/min/1.73 m^2^/year	Urine dipstick ≥1+	Among participants with hematuria at baseline, the proportion with hematuria decreased by 75% (95% CI –87 to –59) at 96 weeks’ treatment
RUBY-3 (NCT05732402), open-label, Madan *et al*., 2024 [77]^a^	Povetacicept	BAFF and APRIL inhibitor	*N* = 41 (enrolled); biopsy-proven IgAN	At 9 months, a 64% reduction in UPCR (interim analysis, *n* = 6)	Not defined	All patients with hematuria^b^ at baseline had hematuria resolution
ENVISION (NCT04287985), randomized, DBPC, Barratt *et al*., 2024 [78]^a^, Mathur *et al*., 2024 [79]	Sibeprenlimab	APRIL inhibitor	*N* = 155; biopsy-proven IgAN	At 12 months, the least-squares mean (±SE) change from baseline in eGFR was –2.7 ± 1.8, 0.2 ± 1.7 and –1.5 ± 1.8 mL/min/1.73 m^2^ in the sibeprenlimab 2 mg, 4 mg and 8 mg groups vs –7.4 ± 1.8 mL/min/1.73 m^2^ in the placebo group	Remission: <5 RBC/HPF	Marked reductions in microscopic hematuria at Months 9, 12 and 16 in those receiving sibeprenlimab vs placebo
ADU-CL-19 (NCT03945318) open-label, Kooienga *et al*., 2025 [80]	Zigakibart	APRIL neutralizing antibody	*N* = 40; biopsy-proven IgAN	At Week 100, 24-h UPCR was reduced by 60.4% from baseline. eGFR remained with an annual eGFR slope (95% CI) of +0.48 (–0.74 to 1.70) mL/min/1.73 m^2^	Positive dipstick analysis	Of patients with hematuria at baseline, 78.6% tested negative at Week 100

a Abstract; ^b^number of patients with hematuria at baseline not reported. DBPC, double-blind, placebo-controlled; SE, standard error; TA, time averaged; UPCR, urine protein:creatinine ratio.

B-cell modulation has been shown to reduce the key components of immune complexes, namely Gd-IgA1 and Gd-IgA1 autoantibodies, along with a concomitant reduction in proteinuria and hematuria (Table 2) [73–80]. In recently completed trials, B-cell modulators that act on the upstream pathophysiology of IgAN (dual inhibitors of BAFF and APRIL, or APRIL only inhibitors) have demonstrated more robust reductions in hematuria. In the Phase 2b ORIGIN trial, hematuria improved to negative/trace in 80% of participants receiving atacicept (a dual BAFF/APRIL inhibitor) compared with 5% receiving placebo at 36 weeks [73]; in the open-label extension period, the proportion of participants with hematuria decreased by 75% at 96 weeks. Similarly, in an open-label study of the APRIL inhibitor zigakibart, 78.6% of participants who had hematuria at baseline tested negative at 100 weeks [80]. These findings have been confirmed in recent clinical trials [34, 35]. These studies suggest that BAFF/APRIL inhibition reduces the ongoing formation of Gd-IgA1-containing immune complexes to permit endogenous resolution of glomerular inflammation, with dipstick hematuria serving as a simple, treatment-sensitive readout at the point of care.

Notably, B-cell modulation results in improvement and stabilization of eGFR [76], unlike previous studies using glucocorticoids where eGFR decline was delayed but not halted, despite the fact that proteinuria reduction exceeded 50% of baseline value. This suggests that the resolution or improvement of inflammation, as manifested by the reduction of hematuria, may be key in achieving preservation of eGFR, even more than proteinuria, and should be incorporated as a therapeutic goal in IgAN treatment [11].

## CONCLUSION AND FUTURE DIRECTIONS

Microscopic hematuria is the most common clinical presentation of IgAN observed in the majority of cases, but it remains an underutilized biomarker. In IgAN, glomerular hematuria may arise during active inflammation due to deposition of immune complexes containing Gd-IgA1 and anti-Gd-IgA1 in the mesangium, which leads to structural damage of the glomeruli. Disease detection and risk-stratification models may be bolstered through the screening of hematuria in addition to proteinuria and eGFR. Furthermore, patients with both proteinuria and hematuria suffer poorer outcomes than those with proteinuria alone [1, 11].

Microscopic hematuria may act as a marker of glomerular inflammation, reflecting therapeutic efficacy, and provides a promising therapy target. While traditional IgAN standard-of-care therapies may have a limited impact on hematuria, clear evidence is lacking because baseline and longitudinal hematuria observations have not been considered in the vast majority of historical studies. However, emerging evidence suggests that B-cell modulation via dual inhibition of BAFF/APRIL or APRIL inhibition may be among the most effective therapies to induce hematuria remission and eGFR stabilization/improvement (Table 2) [73, 74]. This is likely a downstream consequence of a targeted approach early in the pathophysiology of IgAN to decrease production of pathogenic IgA, leading to reductions in immune complex deposition and improvements in glomerular inflammation.

**Figure 3:**
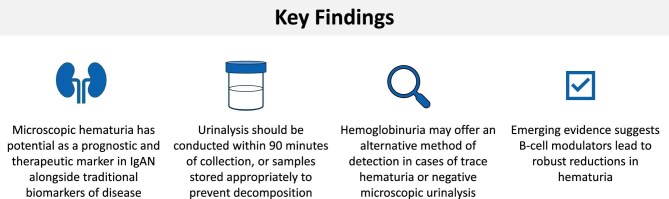
Key findings.

Microscopic hematuria has demonstrated potential as a prognostic and therapeutic marker in IgAN (Fig. 3); however, its full integration into clinical practice requires concerted efforts in following best practice and understanding the value of alternative markers in light of negative urinalysis (i.e. hemoglobinuria), to further highlight its utility in clinical practice. Along with standard measures, such as proteinuria and eGFR, the incorporation of microscopic hematuria/hemoglobinuria monitoring as a biomarker in IgAN will move us away from the “proteinuria-centric” approach that has permeated clinical practice for so long, will improve our understanding of disease pathophysiology, and will help close the gap between mechanistic insight and patient-centered outcomes.

## Data Availability

No new data were generated or analyzed in support of this paper.
